# The Effects of Hydrogen-Rich Water on Blood Lipid Profiles in Metabolic Disorders Clinical Trials: A Systematic Review and Meta-analysis

**DOI:** 10.5812/ijem-148600

**Published:** 2024-09-30

**Authors:** Hamid Jamialahmadi, Ghazaleh Khalili-Tanha, Mostafa Rezaei-Tavirani, Elham Nazari

**Affiliations:** 1 Department of Medical Genetics and Molecular Medicine, School of Medicine, Mashhad University of Medical Sciences, Mashhad, Iran; 2 Metabolic Syndrome Research Center, Mashhad University of Medical Sciences, Mashhad, Iran; 3 Proteomics Research Center, Faculty of Paramedical Sciences, Shahid Beheshti University of Medical Sciences, Tehran, Iran

**Keywords:** Hydrogen-Rich Water, Metabolic Disorders, Lipid Profile, Meta-analysis

## Abstract

**Context:**

Metabolic disorders are a growing global concern, especially in developed countries, due to their increasing prevalence. Serum lipid profiles, including triglycerides (TG), total cholesterol (TC), high-density lipoprotein (HDL), and low-density lipoprotein (LDL), are commonly used clinical biomarkers for monitoring the progression of these metabolic abnormalities. In recent decades, hydrogen-rich water (HRW) has gained attention as a safe and effective treatment, with regulatory effects on lipid peroxidation and inflammatory responses in clinical trials.

**Objectives:**

This systematic review and meta-analysis aim to evaluate the effectiveness of HRW therapy on blood lipid profiles in randomized controlled trials (RCTs) for metabolic disorders.

**Methods:**

Following the PRISMA guidelines, a search for RCT studies was conducted in the PubMed, Web of Science, Embase, and Google Scholar databases up to January 2024. Eight studies that met all eligibility criteria, including RCTs involving metabolic dysfunctions and evaluations of lipid profiles, were included for further analysis. Data extraction was followed by quality evaluation using the Jadad scoring system. Meta-analysis was conducted using STATA software.

**Results:**

The eight selected RCTs included a total of 357 patients with various metabolic disorders. All studies showed either no or low risk of bias. The overall levels of TG [95% CI: -0.27 (-0.47, -0.07)], TC [95% CI: -0.07 (-0.32, -0.18)], and LDL [95% CI: -0.06 (-0.28, 0.15)] demonstrated slight decreases across the studies. However, there was some heterogeneity in HDL levels [95% CI: -0.11 (-0.37, 0.14)] among the studies (I² = 37.32%). Meta-regression analysis further indicated a positive association between the outcomes and the duration of the intervention as a moderating factor.

**Conclusions:**

Hydrogen-rich water demonstrated modest lipid-lowering effects in patients with metabolic disorders. However, due to the observed heterogeneity in HDL variations, further long-term trials involving larger populations are needed to clarify these inconsistencies.

## 1. Context

Metabolic abnormalities and metabolic syndromes are complex conditions that increase the risk of cardiovascular disease (CVD), type 2 diabetes mellitus (T2DM), nonalcoholic fatty liver disease (NAFLD), sleep apnea, polycystic ovarian syndrome (PCOS), lipodystrophies, and premature mortality. The primary risk factors for metabolic disorders include aging, physical inactivity, high blood pressure, insulin resistance, excess body fat around the waist, hormonal imbalances, and dyslipidemia (1-3). Dyslipidemia refers to abnormal lipid levels in the bloodstream, such as elevated levels of triglyceride-rich lipoproteins (TRLs) and low-density lipoprotein (LDL), along with reduced high-density lipoprotein (HDL), all of which are key components of the lipid profile (4). Over the past two decades, the prevalence of metabolic diseases has increased, particularly in developed countries (5). The incidence of metabolic disorders varies across populations and is influenced by factors such as age, gender, and lifestyle (6). For example, the incidence of metabolic syndrome in adults in the United States is estimated to exceed 34% (7). As a result of rapid lifestyle changes and increasingly sedentary habits, the global population faces the growing threat of widespread obesity and type 2 diabetes outbreaks. Consequently, the prevalence of metabolic disorders is expected to rise, especially in developing countries (8).

Hydrogen, the most abundant element in the universe, plays a crucial role in water, organic compounds, and other essential substances (9). Its ability to rapidly diffuse across cellular membranes, combined with its small size and high diffusivity, allows hydrogen to reach areas of the body that may be difficult for other molecules to access. These properties have generated interest in using hydrogen as a potential medical treatment (10). Molecular hydrogen therapy involves various forms of hydrogen delivery, such as hydrogen gas inhalation, H2-rich saline injection, hydrogen water (HW) baths, or hydrogen-rich water (HRW), and is being explored as a preventative and therapeutic option for a variety of conditions, including cancer, CVD, respiratory disease (11), and neurodegenerative disorders (12). Hydrogen demonstrates antioxidant and anti-inflammatory properties, making it potentially useful for reducing oxidative damage and mitigating cellular and mitochondrial harm caused by hydroxyl radicals and oxidized biomolecules (13, 14).

Hydrogen also has anti-inflammatory effects. It can reduce the recruitment of neutrophils and macrophages by inhibiting the expression of chemokines and intercellular adhesion molecules during early-stage inflammation, which leads to decreased production of pro-inflammatory cytokines such as IL-6 and IFN-γ (15).

In recent years, numerous studies have examined the potential benefits of hydrogen therapy on lipid profiles, finding reductions in LDL cholesterol and triglycerides (TG) and increases in HDL cholesterol levels (16, 17). A recent meta-analysis of HRW’s effects on blood lipid profiles in clinical trial populations showed reductions in total cholesterol (TC), TG, and LDL cholesterol after HRW therapy, while HDL levels remained unchanged (18).

## 2. Objectives

In this updated meta-analysis, we focused specifically on clinical trials involving populations with metabolic dysfunctions to summarize the clinical relevance of HRW in managing lipid levels in these patients.

## 3. Methods

### 3.1. Literature Search Strategy and Selection Criteria

This systematic review and meta-analysis adhered to the preferred reporting items for systematic reviews and meta-analyses (PRISMA) guidelines (19). A comprehensive literature search was conducted in PubMed, Embase, Web of Science, and Google Scholar using the following keywords: ("molecular hydrogen" OR "Hydrogen-rich water" OR "HRW") AND ("blood lipid profiles" OR "total cholesterol" OR "high-density lipoprotein cholesterol" OR "low-density lipoprotein cholesterol" OR "lipid") AND ("metabolic disorders" OR "metabolic syndrome" OR "diabetes" OR "T2DM" OR "insulin resistance" OR "impaired glucose tolerance" OR "fatty liver" OR "NAFLD" OR "NASH" OR "obesity" OR "overweight" OR "cardiovascular diseases" OR "stroke" OR "hypertension" OR "high blood pressure" OR "hypercholesterolemia"). Two independent reviewers conducted the literature search, and any disagreements were resolved by consulting a third author. Studies published up to January 2024 were included in this analysis. Additionally, reference lists of related articles and review papers were manually screened to identify further relevant studies.

### 3.2. Inclusion and Exclusion Criteria

To be eligible for inclusion in this meta-analysis, articles had to meet the following criteria: (1) human studies published as original articles; (2) patients with metabolic defects such as fatty liver, hypercholesterolemia, diabetes, insulin resistance, and obesity; (3) a treated group receiving HRW and a control group receiving pure water (PW); (4) double-blinded, randomized, and placebo-controlled trials; (5) studies that included relevant lipid profile indicators (TC, HDL, LDL, and TG).

Exclusion criteria for the meta-analysis were as follows: (1) case reports, reviews, comments, guidelines, in vivo or in vitro studies; (2) other forms of hydrogen therapy, such as inhalation, injection, cream, or eye drops; (3) combination of HRW with other therapies; (4) studies without a placebo group for comparison; and (5) studies lacking pre- and post-treatment data.

### 3.3. Data Extraction

The entire text of each paper was thoroughly screened by two researchers (H.J. and G.K.T.), who independently conducted data extraction. The extracted information from each study included the first author’s name, year of publication, study design, protocol, duration, age of participants, the population size of HRW and PW groups, and outcomes (including means and standard deviations). All literature was carefully reviewed by these two researchers to identify relevant experimental trials for inclusion in the analysis. Any discrepancies or disagreements in the selection process were resolved through discussion between the researchers.

### 3.4. Quality Evaluation of the Included Articles

The Jadad scoring system was employed to assess the methodological quality of the included articles. This evaluation considered eight key features:

1. Randomization: Was the allocation of participants to different groups conducted randomly?

2. Randomization methodology: Was the approach to randomizing participants adequately described and appropriate?

3. Blinding: Was the study designed in a way that both participants and researchers were unaware of group assignments?

4. Blinding methodology: Was the process of maintaining this lack of awareness (blinding) among participants and researchers clearly described and appropriate?

5. Withdrawals and dropouts: Was there an account provided for participants who withdrew or dropped out of the study?

6. Inclusion/exclusion criteria: Were the criteria for selecting or excluding study participants clearly outlined?

7. Adverse effects assessment: Was the procedure for evaluating any negative effects of the intervention clearly described?

8. Statistical analysis methods: Were the techniques used for statistical evaluation of the data clearly articulated?

Each study was evaluated based on these criteria to ensure its quality and reliability in this systematic review and meta-analysis (20).

### 3.5. Statistical Analysis

The STATA software package (version 17.0) was used to perform a meta-analysis using a random effects model and the REML method. The continuous variables of the blood lipid profile are expressed as the mean and standard deviation (SD). The Cochrane Q test and I² index were applied in each meta-analysis to assess study heterogeneity. If P > 0.1 or I^2 ^≤ 50%, it indicated no heterogeneity, and a fixed effects model was used. If P ≤ 0.1 or I^2^ > 50%, it indicated significant research heterogeneity. Forest plots were utilized to visually assess the effect size and the corresponding 95% CI across the studies. Funnel plot distribution was employed to evaluate publication bias in the included literature. A P-value of less than 0.05 was considered statistically significant.

## 4. Results

### 4.1. Literature Search

**Figure 1.**
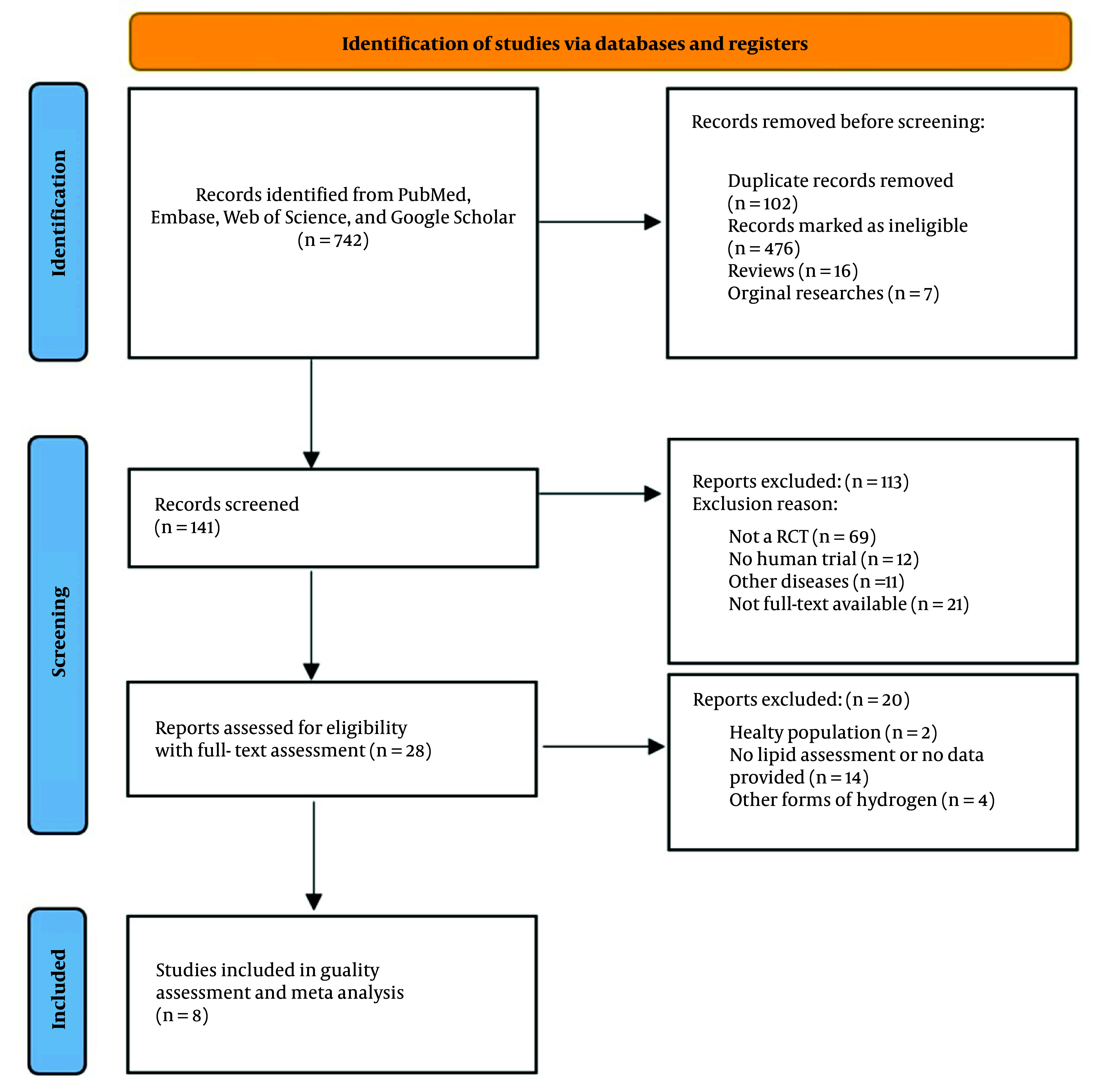
Preferred reporting items for systematic reviews and meta-analyses (PRISMA) flowchart of study selection

The literature search from PubMed, Embase, and Google Scholar identified 742 studies. We found 102 duplicates, and 612 studies were excluded based on a review of the title and abstract. A full-text assessment of the remaining 28 studies led to the exclusion of 20 more studies. Ultimately, 8 studies were included for quality assessment and meta-analysis. The PRISMA flowchart of study selection is shown in Figure 1. 

### 4.2. Study Characteristics

According to the characteristics presented in Table 1, eight randomized control trials were included in this study, involving 357 patients with various metabolic disorders. The patients in three studies had T2DM or impaired glucose tolerance (21-23), two studies involved NAFLD (24, 25), one study focused on metabolic syndromes (26), one study involved hypercholesterolemia (27), and the participants in the final study were overweight women (28).

**Table 1.** Effects of Hydrogen-Rich- Water on Blood Lipid Profiles

Study	Population	Intervention	Outcomes
**1. Kajiyama et al. (** 21 **)**	36 samples with T2DM (58.6 ± 4.7 y)	Study type: Double-blinded, randomized, and placebo-controlled trials. Protocol: 900 mL/d of HRW (H_2_ concentration = 12ppm). Period: 8 weeks	There was no significant difference in the level of HDL-C, LDL-C, RLP-C, TC, and TG between the HRW and PW groups, while the serum level of sdLDL and emLDL significantly decreased in the HRW group. They suggested HRW as a potential supplement in patients with T2DM.
**2. Song et al. (** 27 **)**	68 samples with hypercholesterolemia (55.8 ± 10.5 y)	Study type: Double-blinded, randomized, and placebo-controlled trials. Protocol: 0.9 L/d (0.3 L, three times daily) of HRW. Period: 10 weeks	The levels of LDL, TC, oxidative, and inflammatory markers decreased in plasma, with no significant difference in HDL levels. The findings indicate the potential role of H_2_ in reducing hypercholesterolemia and atherosclerosis.
**3. Korovljev et al. (** 28 **)**	10 women in their middle age who are overweight (56.4 ± 12.6 y)	Study type: Double-blinded, randomized, and placebo-controlled trials. Protocol: A combination of hydrogen-generating minerals (46 mg of calcium and 40 mg of magnesium) provides around six ppm of H_2_ daily. Period: 4 weeks	The TG levels decreased after treatment, while the other blood lipids remained unchanged. The body fat and arm fat rate also dropped in the HRW group. Administration of H_2_ as a blend may be beneficial for managing body composition and insulin resistance in obesity.
**4. LeBaron et al. (** 11 **)**	60 samples with metabolic syndrome (43.2 ± 10 y)	Study type: Double-blinded, randomized, and placebo-controlled trials. Protocol: One tablet thrice a day with 250 mL of water (provide more than 5.5 ppm of H2 daily). Period: 24 weeks	The TC, LDL, VLDL, HDL, TG, glucose levels, and body mass index were decreased in the HRW group after treatment compared with the placebo group.
**5. Kura et al. (** 24 **)**	30 samples with NAFLD (52.9 ± 10.6 y)	Study type: Double-blinded, randomized, and placebo-controlled trials. Protocol: One tablet thrice daily with 330 mL of water (providing more than 4 mg of H_2_ daily). Period: 8 weeks	The HRW significantly reduced BMI and systolic blood pressure while blood lipid profiles remained stable.
**6. Ogawa et al. (** 22 **)**	50 samples with T2DM (68 y)	Study type: Double-blinded, randomized, and placebo-controlled trials. Protocol: H_2_ is administered via EHW. Participants were instructed to consume 1,500 - 2000 mL of water daily, and the electrolyte levels gradually increased over time. Period: 3 months	The EHW group showed a small delta change increasing for TC and HDL and a small decrease for TG compared with the FW group.
**7. Sumbalová et al. (** 25 **)**	30 samples with NAFLD (52.9 ± 2.7 y)	Study type: Double-blinded, randomized, and placebo-controlled trials. Protocol: One tablet thrice daily with 330 mL of water (providing more than 4 mg/L of H_2_ daily). Period: 8 weeks	The HDL cholesterol (+7.2%) and immune cells (lymphocytes) concentration was enhanced in the HRW group, while TC and TG slightly increased.
**8. Liang et al. (** 23 **)**	73 samples with IFG (47 ± 5.73 y)	Study type: Double-blinded, randomized, and placebo-controlled trials. Protocol: One L/d of HRW (H_2_ concentration = 1.4 mg/mL), Period: 8 weeks	Subjects showed a decreasing trend in TC following HRW treatment, with no significant difference in HDL and LDL levels.

Abbreviations: HDL, high-density lipoprotein; LDL, low-density lipoprotein; BMI, Body Mass Index; TC, total cholesterol; TG, triglycerides; HRW, hydrogen-rich water; PW, placebo water; T2DM, type 2 diabetes; IFG, impaired fasting glucose; NAFLD, non-alcoholic fatty liver disease; HDL-C, high-density lipoprotein cholesterol; LDL-C, low-density lipoprotein cholesterol; RLP-C, remnant-like particle cholesterol; sdLDL, small dense LDL; emLDL, net electronegative charge of modified LDL.

Participants in the treated groups of all studies received HRW, ranging from 750 mL to 2000 mL per day, with six studies using 900 to 1000 mL/day (21, 23-25, 27, 28), one study using 750 mL/day (26), and one study using 1500 - 2000 mL/day (22). Hydrogen concentration data was provided in only four studies (21, 24-26). The trial periods ranged from 4 weeks (28) to 12 weeks (22), while most studies used an 8-week follow-up trial (21, 23-25).

All analyzed blood lipid variables, including TC, TG, HDL, and LDL, were assessed in six studies (21, 23, 24, 26-28). However, two studies (22, 25) did not assess blood LDL levels. More detailed characteristics of the lipid profiles from the included studies are provided in Table 2. 

**Table 2.** Basic Information and the Related Blood Lipids Profile of the Included Studies

Author; (y)	Research Object	Total Cases		BMI (kg/m^2^)		TC (mmol/L)		LDL (mmol/L)		HDL (mmol/L)		TG (mmol/L)	
		**HRW**	**PW**	**HRW**	**PW**	**HRW**	**PW**	**HRW**	**PW**	**HRW**	**PW**	**HRW**	**PW**
**Kajiyama et al. (2008), (** 21 **)**	T2DM	36	36	23.5 ± 3.3	23.5 ± 3.3	5.45 ± 0.87	5.5 ± 0.92	1.63 ± 0.37	1.6 ± 0.37	3.35 ± 0.73	3.42 ± 0.78	10.8 ± 4	11.3 ± 4.4
**Song et al. (2015), (** 27 **)**	Hyperchol Esterolemia	34	34	26.9 ± 3.6	26.7 ± 3.3	5.65 ± 1.3	6.09 ± 1.18	1.45 ± 0.36	1.51 ± 0.32	3.85 ± 1.21	4.08 ± 1.15	1.85 ± 1.17	2 ± 1.2
**Korovljev et al . (2017), (** 28 **)**	Obesity	10	10	29.2 ± 3.2	29.6 ± 3.4	5.7 ± 1	5.9 ± 0.8	1.5 ± 0.2	1.6 ± 0.4	3.6 ± 0.7	3.7 ± 0.7	1.2 ± 0.6	1.4 ± 0.7
**LeBaron et al. (2020), (** 11 **)**	MetS	30	30	28.2 ± 4.9	31.3 ± 5.3	9.39 ± 1.45	10.2 ± 2.14	2.24 ± 0.1	2.34 ± 0.13	5.69 ± 1.55	5.88 ± 43.3	7.91 ± 3.61	10.3 ± 5.62
**Kura et al. (2022), (** 24 **)**	NAFLD	17	13	35.16 ± 4.33	32.91 ± 3.03	10.5 ± 2.15	10.43 ± 2.28	2.58 ± 0.22	46.05 ± 8.7	6.69 ± 1.7	6.66 ± 1.7	9.41 ± 4.13	10.86 ± 3.86
**Ogawa et al. (2022), (** 22 **)**	T2DM	23	22	25.9 ± 3.8	27.7 ± 5	5.3 ± 0.83	4.75 ± 0.66	1.34 ± 0.43	1.25 ± 0.55	-	-	1.21 ± 0.42	1.15 ± 0.51
**Sumbalová et al. (2023), (** 25 **)**	NAFLD	17	13	35.2 ± 1.1	32.9 ± 0.8	4.87 ± 0.24	4.86 ± 0.29	1.19 ± 0.03	1.19 ± 0.06	-	-	1.97 ± 0.2	2.21 ± 0.22
**Liang et al. (2023), (** 23 **)**	IFG	32	41	25.09 ± 4.15	24.63 ± 3.36	5.11 ± 0.73	5.22 ± 0.96	1.24 ± 0.32	1.22 ± 0.27	3.13 ± 0.61	3.11 ± 0.74	1.14 ± 1	1.42 ± 1.05

Abbreviations: BMI, Body Mass Index; TC, total cholesterol; TG, triglycerides; HRW, hydrogen-rich water; PW, placebo water; T2DM, type 2 diabetes; IFG, impaired fasting glucose; NAFLD, non-alcoholic fatty liver disease; MetS, metabolic syndrome.

### 4.3. Quality Assessment

To evaluate the quality of the included studies, a Jadad quality score was applied. According to this scoring, as shown in Table 3, all studies received scores ranging from 4 to 8, indicating that these studies exhibited "good" to "excellent" methodological quality.

**Figure 2.**
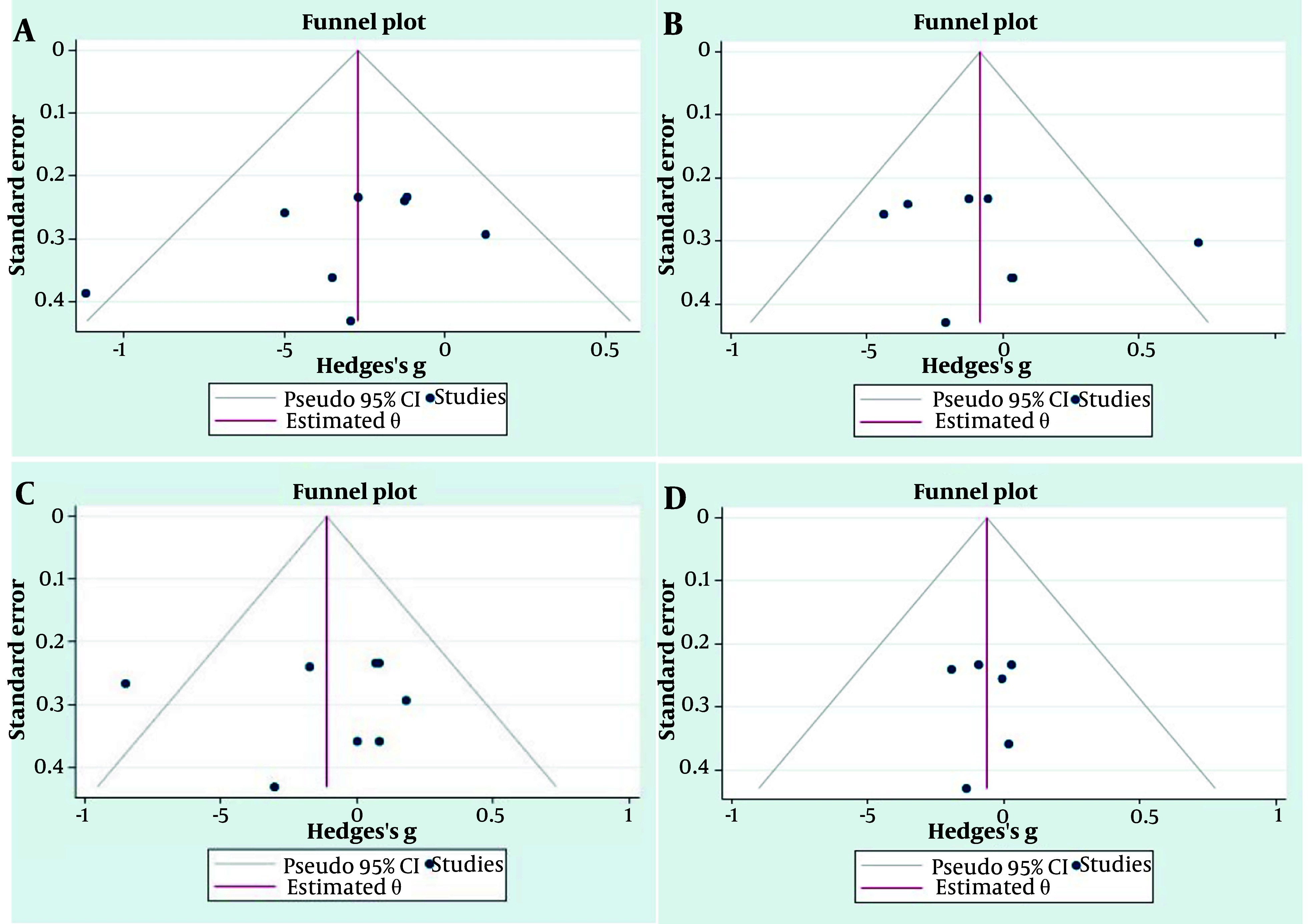
Publication bias assessment with Hedges’s method for blood lipid profiles. The A, B, C, and D funnel plots represent the TG, TC, HDL, and LDL respectively

Additionally, publication bias for all variables was assessed using Hedges’s method, with the results illustrated in Figure 2. 

**Table 3.** Jadad Quality Control Scoring of the Included Studies

Questions (Yes = 1; No = 0)	Kajiyama et al. (21)	Liang et al. (23)	Korovljev et al. (28)	Sumbalová et al. (25)	Kura et al. (24)	LeBaron et al. (11)	Song et al. (27)	Ogawa et al. (22)
**Was the study described as randomized?**	1	1	1	1	1	1	1	1
**Was the method of randomization appropriate?**	0	0	0	1	1	1	1	1
**Was the study described as blinding?**	1	1	1	1	1	1	1	1
**Was the method of blinding appropriate?**	0	0	0	0	0	0	1	1
**Was there a description of withdrawals and dropouts?**	0	1	0	0	0	0	0	1
**Was there a clear description of the inclusion/exclusion criteria?**	1	1	0	1	1	1	1	1
**Was the method used to assess adverse effects described?**	0	0	1	0	0	0	1	1
**Were the methods of statistical analysis described?**	1	1	1	1	1	1	1	1
**Total Score**	4	5	4	5	5	5	7	8

### 4.4. Effects of Hydrogen-Rich Water on Blood Lipid Profile

We evaluated the effects’ size with a random effects reml model due to better assessment of heterogenous studies, however, due to the limited studies, we checked the heterogeneity of studies with a fixed effects model, and totally no obvious differences were observed between the two models.

#### 4.4.1. Triglycerides

**Figure 3.**
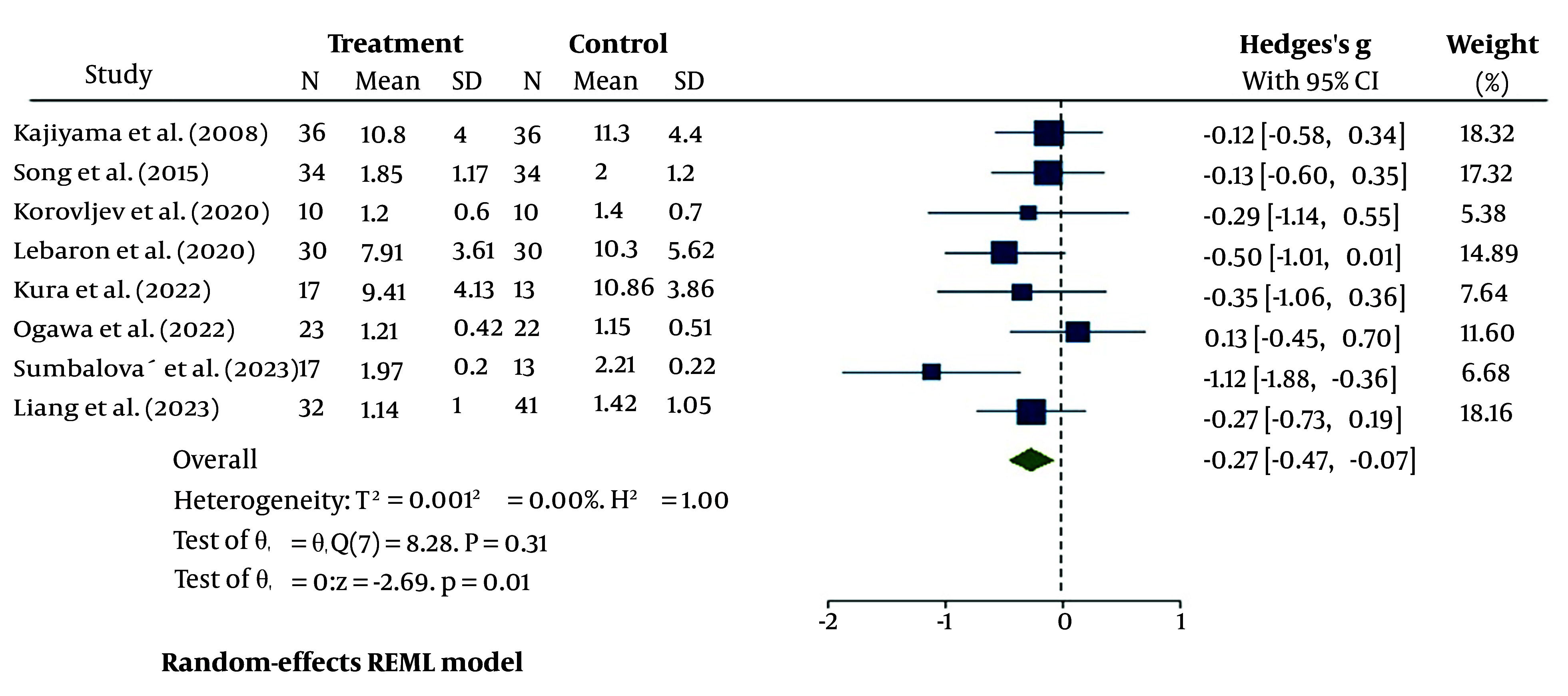
The heterogeneity assessment of the eight included studies for serum triglycerides (TG)

Triglycerides results from the eight studies indicated a slight decrease in HRW-treated patients compared to the PW control group [95% CI: -0.27 (-0.47, -0.07)]. No heterogeneity bias was detected in the analysis of these studies (I^2^ = 0%, P = 0.31) (Figure 3). 

#### 4.4.2. Total Cholesterol

**Figure 4.**
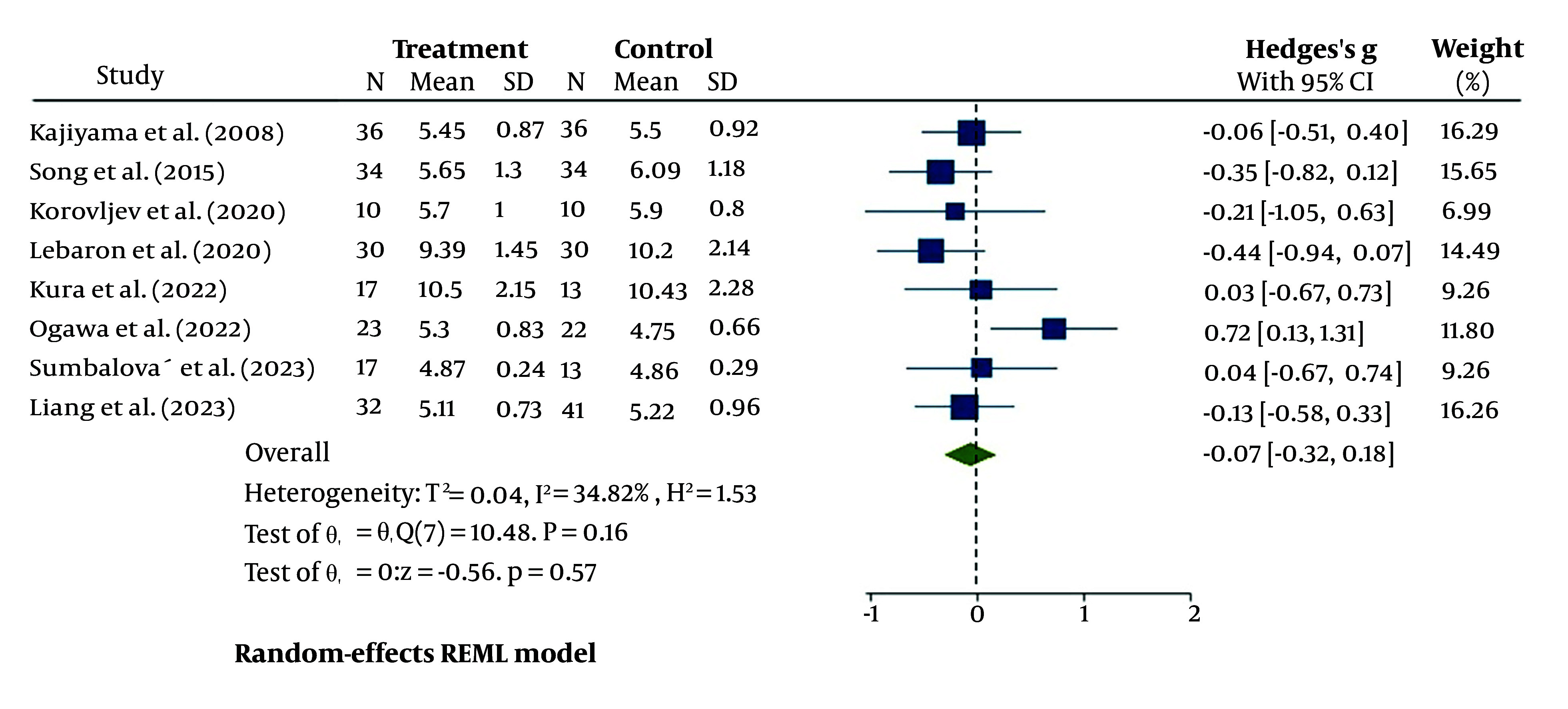
The forest plot for heterogeneity assessment of studies for serum total cholesterol (TC)

The changes in TC after treatment with HRW were not significant compared to the control group, with a 95% CI of -0.07 (-0.32, -0.18); however, this treatment did show a small decreasing effect on TC (Figure 4). Additionally, the heterogeneity analysis revealed moderate heterogeneity, with I^2^ = 34.82% and P = 0.16.

#### 4.4.3. High-Density Lipoprotein

**Figure 5.**
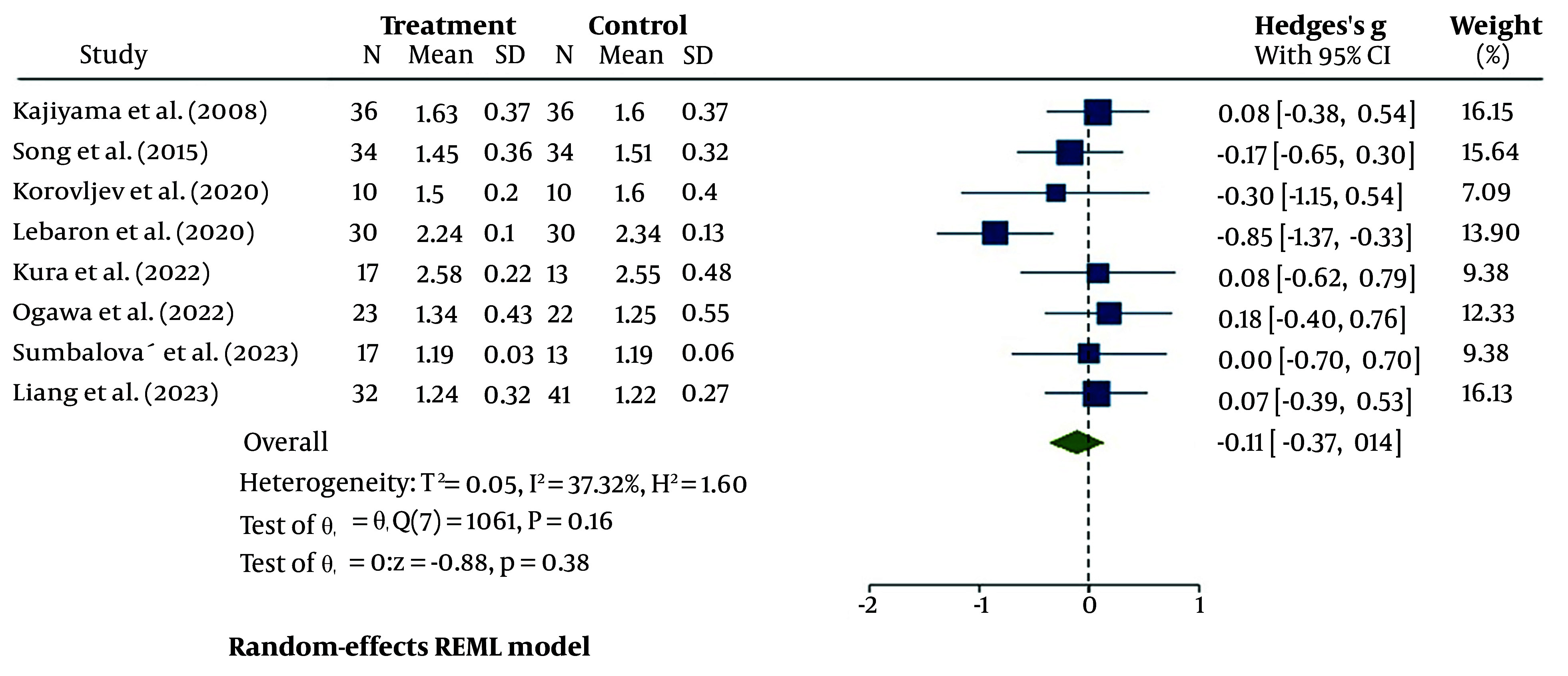
The forest plot for heterogeneity assessment of studies for serum high-density lipoprotein (HDL)

**Figure 6.**
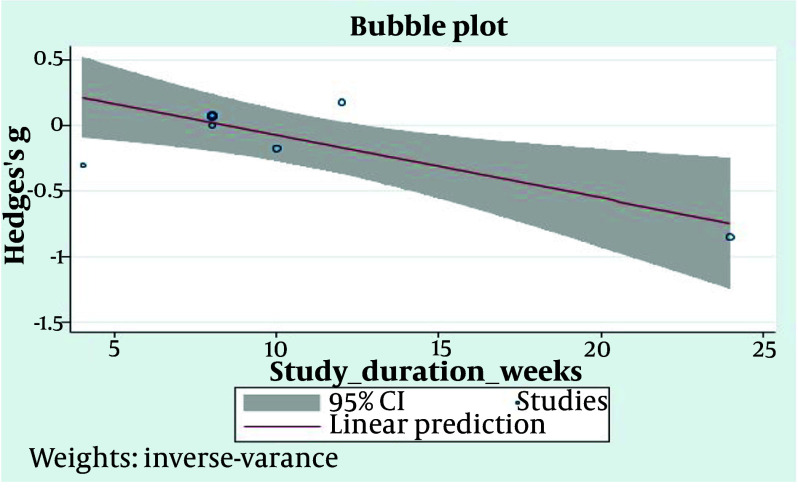
The bubble plot for assessment of studies durations regression for serum high-density lipoprotein (HDL)

As shown in Figure 5, the evaluation of eight pooled studies for serum HDL changes after treatment with HRW revealed a small decline compared to the control [95% CI = -0.11 (-0.37, 0.14)]. The heterogeneity assessment indicated moderate heterogeneity (I^2^ = 37.32%). Additionally, when using a fixed-effects model, the heterogeneity remained relatively unchanged (I^2^ = 34%) (Figure 6). 

#### 4.4.4. Low-Density Lipoprotein

**Figure 7.**
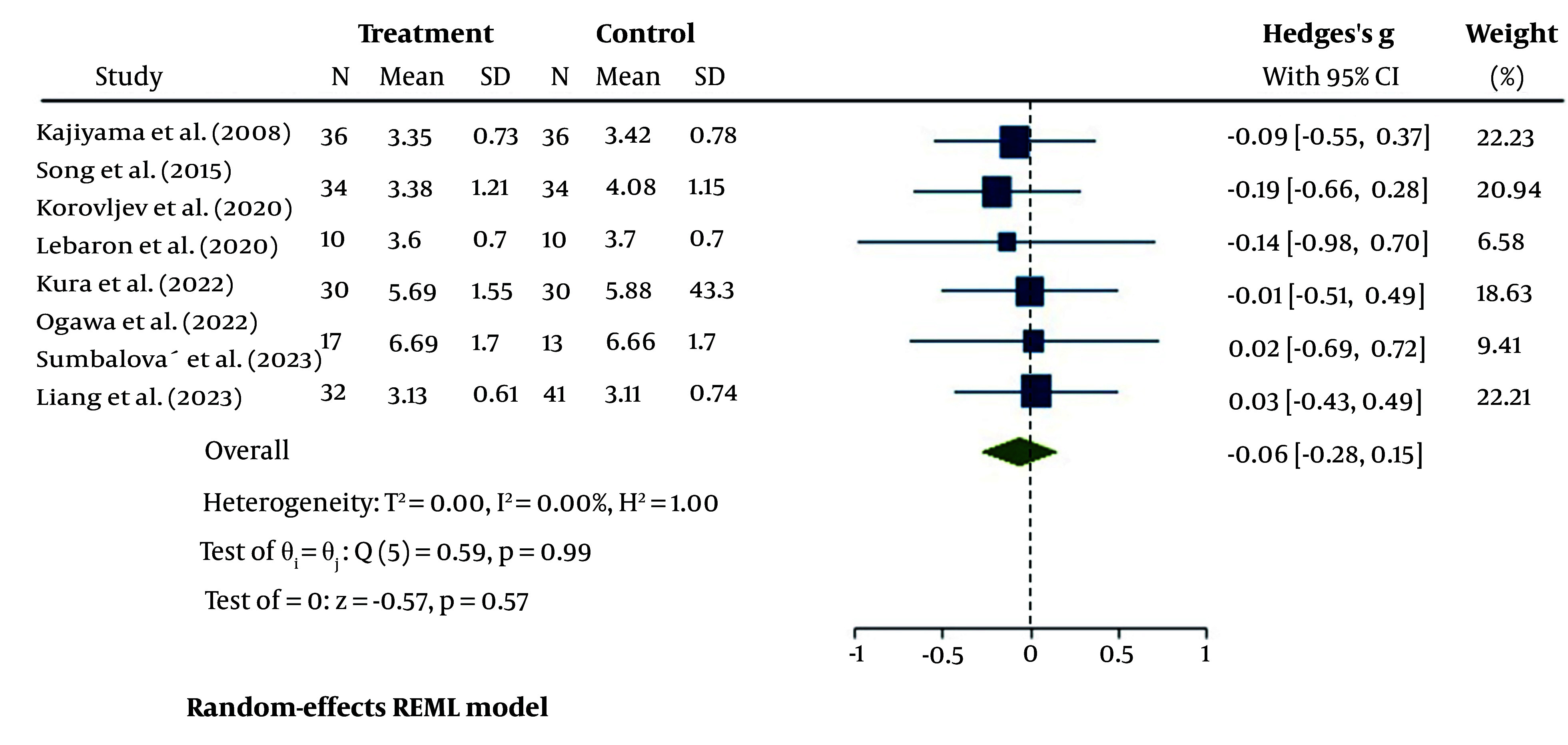
The forest plot for heterogeneity assessment of studies for serum low-density lipoprotein (LDL)

The overall results of the eight studies were pooled using a random-effects model to analyze the changes in blood LDL after treatment with HRW compared to PW controls (Figure 7). According to the results, the pooled studies showed no heterogeneity (I^2^ = 0%, P = 0.99). Although HRW treatment had a small decreasing effect on LDL levels, the changes were not statistically significant [95% CI = -0.06 (-0.28, 0.15)].

## 5. Discussion

In the current systematic review and meta-analysis, we focused on evaluating the effects of HRW on lipid profile changes in various metabolic disorders through a review of the literature. After rigorous screening of trial studies up to 2024, eight double-blind RCTs, involving 357 patients with different metabolic disorders, were selected for further analysis. The assessment of serum lipid changes in all eight studies showed that TG, TC, HDL, and LDL levels slightly decreased following HRW therapy. However, most of these changes were not statistically significant.

Metabolic disorders are a complex set of metabolic or endocrine disturbances that lead to various conditions, including metabolic syndrome (MetS), obesity, CVD, T2DM, impaired glucose tolerance, non-alcoholic fatty liver disease (NAFLD), and hypercholesterolemia (29). These disorders have become a global concern due to lifestyle changes and other risk factors. Although the prevalence of these disorders is high among older adults, there has been an increasing trend among younger adults aged 20 - 30, as observed up to 2016 in the United States (7). Alterations in lipid profiles and their metabolism in tissues are major consequences of metabolic dysfunctions, leading to increased synthesis of VLDL and TG in the liver while decreasing HDL levels. Furthermore, TG levels in plasma rise due to reduced TG uptake by peripheral tissues. Another consequence of metabolic disorders is the disruption of the endocrine system, which leads to hormonal changes, such as elevated insulin and leptin levels, insulin resistance in peripheral tissues, and decreased adiponectin, all of which contribute to reduced fatty acid oxidation (30).

Hydrogen therapy has demonstrated several regulatory effects, including antioxidation, anti-inflammation, and suppression of apoptosis (31). Some studies have shown that hydrogen therapy decreases serum TC, TG, and LDL levels while increasing HDL. It also reduces isocitrate lyase activity and the glyoxylic acid cycle. Additionally, H_2_ consumption can affect lipid oxidation by increasing superoxide dismutase levels and reducing thiobarbituric acid-reactive substances (32).

A study by Todorovic et al. assessed the effects of HRW on lipid profiles in a pool of studies up to 2022, focusing on clinical trial populations with metabolic abnormalities and physiological issues such as aging. Their findings revealed a decrease in TC, TG, and LDL levels following treatment in all their included studies, while total HDL remained unchanged (18). Unlike their study, we limited our analysis to serum lipid profile changes in metabolic disorders in double-blind RCTs, incorporating updates from more recent studies. Our study revealed differing effects of HRW on serum HDL compared to Todorovic et al. (18).

Furthermore, we considered potential moderators, including age, nationality, intervention period, and doses that may have influenced the outcomes, and performed a meta-regression analysis. However, since some data were not provided—such as information on the nationality or age of participants—we primarily assessed the effects of intervention periods. All studies included participants of both sexes, except for Korovljev et al., which exclusively involved overweight women. As a result, subgroup analysis was not performed (28).

The total trend of TG, a major hyperlipidemia factor, showed a reduction after hydrogen therapy in these studies. However, the study by Ogawa et al. did not demonstrate any reduction in serum TG. Although they did not provide an explanation, one possible reason may be the different effects of electrolyzed hydrogen water (EHW) compared to HRW used in other studies (22). The meta-regression test showed no significant association between the intervention period and changes in TG, TC, and LDL.

Hydrogen-rich water generally had a slight decreasing effect on TC, a well-known predictor of CVD, metabolic disorders, and hyperlipidemia, although this effect was not significant (P = 0.57). In a study conducted by LeBaron et al., this decrease was more noticeable, possibly due to the higher doses of H_2_ (5.5 ppm) administered and the longer treatment period (24 weeks). However, the regression analysis did not support any association between TC variations and the duration of treatment. Conversely, in some studies, TC slightly increased. In the study by Ogawa et al., TC slightly increased, possibly due to the administration of EHW, although the authors did not discuss the possible reasons, stating only that EHW had no adverse effects, such as increased H+ levels or hyperkalemia (22, 26).

High-density lipoprotein is a predictive biomarker for the risk of coronary artery disease (CAD) due to its protective roles, including cholesterol transfer from peripheral tissues to the liver for excretion through bile, absorption of cholesterol from macrophage foam cells, anti-inflammatory and antioxidant activity, and detoxification of lipid hydroperoxides. These functions make HDL a risk factor for atherosclerosis and other vascular diseases (33, 34). Similar to other clinical trials, Nakao et al. reported in a pilot study with MetS patients a significant increase in HDL and a subsequent decrease in the TC/HDL ratio after 4 weeks of HRW treatment (16, 35).

Our assessment of HDL changes after HRW therapy in the selected studies showed that the total trend was not an increase but rather a slight decrease, though this decrease was not statistically significant (P = 0.38). It is important to note that in three studies, the 95% CI was negative. We thoroughly examined the possible reasons for these variations. The meta-regression analysis on study duration showed a positive correlation between this potential moderator and the study results, with R2 = 100% and P < 0.05. The study by LeBaron et al., which showed the largest decrease in HDL levels, demonstrated a significant decline in the ratio of TC or TG to HDL, both of which are considered more important predictive biomarkers due to the significant reduction in TC and TG (26).

Furthermore, in a clinical trial, Song et al. found that plasma HDL-C increased after 10 weeks of HRW administration. The authors indicated that HRW led to an increase in nascent HDL forms, including pre-β1-HDL, and improved HDL functions, such as cholesterol efflux by the ABCA1 transporter, reduced phospholipid content in HDL3, and enhanced apoM in HDL particles, which is involved in restricting atherogenesis (27). Reviewing another study revealed that despite a decline in serum TG levels after a 4-week HRW intervention, other lipid levels did not change significantly. However, a closer analysis of the provided data indicated a small decrease in both the treatment and placebo groups (28).

The last serum lipid marker evaluated in this analysis, LDL, is another key biomarker for hyperlipidemia and coronary heart disease (CHD), alongside other factors such as small dense LDL (sd-LDL), non-high-density lipoprotein cholesterol (non-HDL-c), and Apolipoprotein B (ApoB) (36, 37). However, due to insufficient data on other LDL subtypes and lipids in the eight selected studies, our analysis focused solely on LDL. Overall, after the interventions, the serum LDL levels slightly decreased. Two of the studies did not provide LDL data (22, 25), and in two studies, LDL levels either slightly increased or showed a marginal decrease (23, 24). In the study conducted by Kura et al., the delta change in LDL showed a decrease in both the treated and untreated groups. They suggested that the limited study duration (8 weeks compared to 24 weeks in their previous study) and the higher baseline parameters in the earlier study could account for these results (24).

In Liang et al.'s study, there were no significant changes in LDL or HDL levels. The authors attributed this to the short treatment duration (8 weeks) and the small sample size, suggesting that a longer-term trial could clarify these ambiguities (23).

This meta-analysis focused on populations with metabolic disorders but had some limitations, including the small number of RCT studies and insufficient data regarding changes in patients' lipid profiles. Furthermore, some studies did not provide comprehensive lipid profile information for the patients.

### 5.1. Conclusions

In conclusion, HRW therapy shows potential as a regulatory factor for plasma lipid profiles. However, the overall effects were modest, and HRW may be more effective when combined with lifestyle modifications and other treatments. The heterogeneity of the treatment period impacted the outcomes for HDL, with longer intervention periods resulting in more effective lipid-lowering effects. Nevertheless, many studies lacked clear data on the administered doses, a critical factor that could influence the outcomes. According to our data, no publication bias was observed due to the strict inclusion criteria and review of RCT studies, although this limited the populations studied.

## Data Availability

The dataset presented in the study is available on request from the corresponding author during submission or after publication.
